# Extension domain of amyloid processor protein inhibits amyloidogenic cleavage and balances neural activity in a traumatic brain injury mouse model

**DOI:** 10.1111/cns.14402

**Published:** 2023-08-17

**Authors:** Zhenxing Xie, Tianyu Li, Wei Su, Yanyun Lou, Yongsheng Zhang, Xiyuan Zhou, Zhanfei Li, Xiangjun Bai, Xinghua Liu

**Affiliations:** ^1^ Division of Trauma Surgery, Emergency Surgery & Surgical Critical, Tongji Trauma Center, Tongji Hospital, Tongji Medical College Huazhong University of Science and Technology Wuhan China; ^2^ Department of Emergency and Critical Care Medicine, Tongji Hospital, Tongji Medical College Huazhong University of Science and Technology Wuhan China

**Keywords:** APP, behaviors, GABAergic neurons, neural activity, sAPP, TBI

## Abstract

**Background:**

Mechanisms underlying cognitive dysfunction following traumatic brain injury (TBI) partially due to abnormal amyloid processor protein (APP) cleavage and neural hyperactivity. Binding of the extension domain of APP (ExD17) to the GABAbR1 receptor results in reduced neural activity, which might play a role in the mechanisms of cognitive dysfunction caused by TBI.

**Methods:**

Stretch‐induced injury was utilized to establish a cell injury model in HT22 cells. The TBI model was created by striking the exposed brain tissue with a free‐falling weight. Topical or intraperitoneal administration of ExD17 was performed. Cell viability was assessed through a cell counting kit‐8 assay, while intracellular Ca^2+^ was measured using Fluo‐4. Western blotting was used to investigate the expression of APP amyloidogenic cleavage proteins, GABAbR1, phospholipase C (PLC), PLCB3, and synaptic proteins. ELISA was performed to analyze the levels of Aβ42. Seizures were assessed using electroencephalography (EEG). Behaviors were evaluated through the novel object recognition test, open field test, elevated plus maze test, and nest‐building test.

**Results:**

ExD17 improved cell viability and reduced intracellular calcium in the cell injury model. The treatment also suppressed the increased expression of APP amyloidogenic cleavage proteins and Aβ42 in both cell injury and TBI models. ExD17 treatment reversed the abnormal expression of GABAbR1, GRIA2, p‐PLCG1/PLCG1 ratio, and p‐PLCB3/PLCB3 ratio. In addition, ExD17 treatment reduced neural activity, seizure events, and their duration in TBI. Intraperitoneal injection of ExD17 improved behavioral outcomes in the TBI mouse model.

**Conclusions:**

ExD17 treatment results in a reduction of amyloidogenic APP cleavage and neuroexcitotoxicity, ultimately leading to an improvement in the behavioral deficits observed in TBI mice.

## Graphical abstract

The extension domain of amyloid processor protein (APP), ExD17, reduces amyloidogenic APP cleavage in traumatic brain injury (TBI) mice. ExD17 inhibits neuroexcitotoxicity by regulating the expression of GABAbR and glutamate receptors in TBI mice.

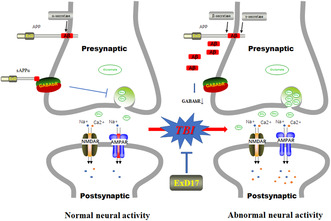

## 1 INTRODUCTION

Several epidemiological studies have provided compelling evidence that traumatic brain injury (TBI) is associated with an increased risk of degenerative neurocognitive conditions resulting in dementia.^1^, ^2^, ^3^, ^4^, ^5^ Many studies suggest that the etiology of cognitive impairment following TBI shares similarities with that of Alzheimer's disease (AD).^6^, ^7^ The accumulation of amyloid plaques is a common pathological feature observed in both TBI and AD patients.^8^ Amyloid plaques, mainly consist of Aβ40 and Aβ42, are produced by amyloidogenic cleavage of amyloid processor protein (APP), in which APP is first cleaved by beta‐secretase followed by γ‐secretase. The accumulation of amyloid plaques in TBI brains suggests that APP cleavage is aberrantly activated during disease progression. Besides the neurotoxic amyloid β‐protein (Aβ) peptides, APP can also be cleaved into various secreted APP variants. In a normal functioning nonamyloidogenic pathway, in brief, APP is cleaved by α‐secretase to release sAPP‐α, which is further cleaved by γ‐secretase to produce a truncated version of Aβ.^9^ Rice et al. demonstrated that the interaction between sAPP‐α and the sushi domain of GABAbR1a plays a role in modulating synaptic transmission and inhibiting neural activity.^10^ Within APP, the GABAbR1a binding domain could be narrowed down to 17 evolutionarily conserved amino acids in the extension domain (ExD17). This discovery offers valuable information about the physiological role of APP and potential avenues to improve cognitive function in TBI patients not only by promoting nonamyloidogenic pathways but also by targeting Aβ processing.

After TBI in rats, the ipsilateral thalamus of the injured hemisphere shows a decrease in GABAb receptor expression.^11^ The reduction of GABAb receptor expression and function in TBI contributes to severe TBI sequelae, such as epilepsy.^12^ The imbalance of cleavage products of APP may disrupt the balance of brain excitation and inhibition, and interact abnormally with GABA receptors in TBI, potentially leading to cognitive dysfunction in addition to cell death from imbalanced neural activity and traumatic neurons.

In our study, we hypothesized that sAPP deficiency would lead to neural hyperactivity and the formation of amyloid plaque in a mouse model of TBI. The administration of a valid segment of sAPP, ExD17, could reduce abnormal neural activity, compete with APP amyloidogenic cleavage, and improve behavioral outcomes in the TBI mouse model.

## 2 METHODS AND MATERIALS

### 2.1 Peptides

**FIGURE 1:**
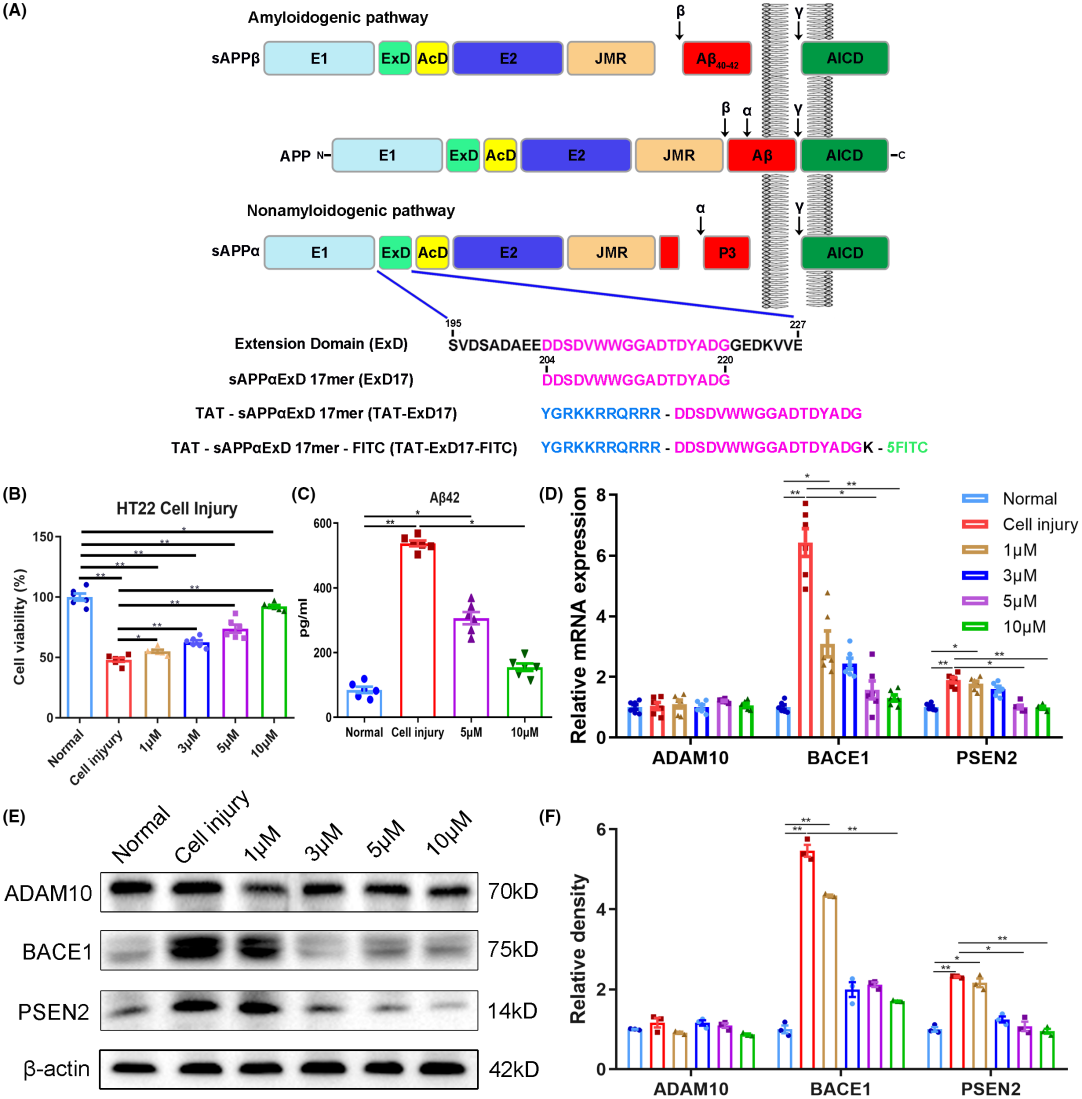
ExD17 inhibits APP amyloidogenic cleavage in a model of HT22 cell injury. (A) Schematic diagram illustrating the cleavage of APP and representation of peptides. In amyloidogenic cleavage, β/γ‐secretase cleavage yields Aβ40 and Aβ42, whereas nonamyloidogenic cleavage yields p3 via α/γ‐secretase cleavage. ExD17 is a peptide of 17 amino acids in the extension domain, and TAT is added to the N‐terminus of ExD17. FITC is added to the C‐terminus of ExD17 via lysine for detection. (B) The effect of ExD17 on cell viability was assessed by CCK8 assay. HT22 cells were treated with various concentrations of ExD17 for 24 h after injury. *N* = 6 wells per group. (C) ELISA assessment of Aβ42 levels in the culture medium of control, injury, and injury treated with various concentrations of ExD17 for 24 h. *N* = 6 wells per group. (D) RT‐PCR was performed to assess mRNA expression levels of ADAM10, BACE1, and PSEN2 24 h after injury and treatment with various concentrations of ExD17. *N* = 6 wells per group. (E and F) Western blot analysis and statistical analysis of ADAM10, BACE1, and PSEN2 expression levels after cell injury and treatment with various concentrations of ExD17. *N* = 3 wells per group. Values are presented as means ± SEM. *p < 0.05, **p < 0.01.

The peptide sequences were synthesized by ChinaPeptides Co., Ltd. The TAT moiety is YGRKKRRQRRR, corresponding to amino acids 47–57 of the HIV TAT protein, while the ExD17 moiety is DDSDVWWGGADTDYA, corresponding to amino acids 204–220 of human/mouse APP. The peptide sequences for ExD17, TAT‐ExD17, and TAT‐ExD17‐FITC are DDSDVWWGGADTDYA, YGRKKRRQRRR‐DDSDVWWGGADTDYA, and YGRKKRRQRRR‐DDSDVWWGGADTDYADGK‐FITC, respectively (Figure 1A).

### 2.2 Cell culture and stretch injury

Mouse hippocampal cells (HT‐22) were purchased from American Type Culture Collection and cultured in Dulbecco's modified Eagle's medium (Gibco) supplemented with 10% fetal bovine serum (40130ES76, YEASEN), 100 μg/mL penicillin, and 100 μg/mL streptomycin in a humidified incubator (Heraeus) with 5% CO_2_. For cell injury, cells were seeded at 1 × 10^5^/mL in Bioflex culture plates (FlexCell International) and incubated for 24 h. Moderate stretch injury was applied using the cell injury controller II (CIC; Custom Design & Fabrication), which exerted a controlled pulse of compressed nitrogen gas to transiently deform the silastic membrane and to achieve a predetermined degree of stretch.^13^ The parameters for the moderate stretch injury were set at 28 psi (pounds per square inch) with a 50‐ms duration, resulting in 10.2 psi peak pressure at the well. After the cell injury, cells were further incubated for 24 h before conducting the subsequent biological experiments. For ExD17 treatment, ExD17 was prepared in 17% acetonitrile/83% ddH_2_O as a stock solution with a concentration of 500 μM. The ExD17 was added to the culture medium immediately after cell injury at concentrations of 1, 3, 5, and 10 μM.

### 2.3 Measurement of cell viability

To measure cell viability, a CCK‐8 assay kit (HY‐K0301, MCE) was used 24 h after injury according to the manufacturer's instructions. The cells in 6‐well plates were treated with 10% CCK‐8 solution and incubated at 37°C for 1.5 h. Subsequently, 100 μL of supernatant per well was transferred to a 96‐well plate and the optical density (OD) was measured at 450 nm using a microplate reader (Multiskan FC, Thermo).

### 2.4 Measurement of intracellular Ca^2+^

Intracellular Ca^2+^ was measured by Fluo 4‐AM (40704ES50, Yeasen), a membrane‐permeable fluorescent indicator for Ca^2+^. Briefly, HT22 cells were washed three times with Ca^2+^ and Mg^2+^‐free Hanks' balanced salt solution (HBSS) and loaded with Fluo 4‐AM (4 μM) for 40 min at 37°C in HBSS. The cells were then washed three times with HBSS and imaged using a fluorescence microscope equipped with a CCD camera (Mshot).

### 2.5 Animals

Wild‐type male C57BL/6 mice of 10–12 weeks of age were assigned randomly into each of the three experimental groups: sham (mice that underwent a craniotomy without weight‐drop injury), TBI + vehicle control (abbreviated as TBI group, mice that underwent TBI and were given saline), and TBI + ExD17 (abbreviated as ExD17 group, mice that underwent TBI and were given ExD17). The experimental protocols were approved by the Experimental Animal Ethical Committee of Tongji Hospital affiliated with Huazhong University of Science and Technology.

### 2.6 Establishment of TBI model and ExD17 administration

The mice were anesthetized with pentobarbital sodium (50 mg/kg intraperitoneally, i.p.) and fixed to a stereotactic instrument. A 3‐mm‐diameter craniotomy was performed on the left parieto‐temporal cortex, with the bone flap removed, while the dura was left intact. To establish the TBI model, a 40‐g weight was free fell from a height of 20 cm to hit the dura.

To achieve topical delivery of ExD17 directly onto the brain, 1.5 or 2.5 μL of 250 μM ExD17 in saline was applied using a pipette. After approximately 5 min, the scalp was sutured once the fluid had penetrated the cortex. On postoperative Day 3, the mice underwent behavioral tests and were subsequently killed.

To examine the effect of i.p. administration of ExD17, TAT‐ExD was injected at doses of 10 or 20 mg/kg at 0, 24, and 48 h after TBI surgery. To identify whether TAT‐ExD could cross the blood–brain barrier, 20 mg/kg TAT‐ExD‐FITC was i.p. injected and the brains were collected at 6 and 24 h postinjection.

### 2.7 Tail suspension test

Behavioral tests were conducted sequentially in the following order: nest‐building test, open field test, novel object recognition test (NORT), and elevated plus maze test (EPMT). The tail suspension test, as described in previous studies,^14^ was performed 3 days after surgery. The mice were suspended by adhesive tape located approximately 1 cm from the tip of the tail, 50 cm above the floor, for 6 min. Immobility was defined as the cessation of struggling of the body and paws, and the duration of immobility during the last 4 min was recorded.

### 2.8 Open field test

On the third day after the surgery, the mice were placed individually in the middle of a square arena (40 cm in height, 50 cm in width and length) with a camera mounted above. The locomotion was assessed by measuring the total distance traveled (cm) and average velocity (cm/s) during the 5‐min trial. The arena is divided into 25 equal parts, and the time spent in the central nine parts was considered as the time spent in the center. The arena was cleaned thoroughly with 75% alcohol after each trial.

### 2.9 Novel object recognition test

One hour after the open field test (OFT), mice were subjected to the arena again, which contained two identical objects, and the mice were allowed to freely explore for 10 min. After a 1‐h interval, mice were placed back into the arena, in which one object was replaced with a novel object, and allowed to explore for 10 min. The objects and the field were cleaned with 75% ethanol between each trial. The time that mice interacted with objects was recorded. The discrimination index = (the time spent on the novel object−the time spent on the familiar object)/total time spent on both objects × 100.

### 2.10 Nest‐building behavior test

Nest‐building behavior tests were carried out according to previous studies.^15^ The nest‐building task is a natural motor behavior requiring the use of orofacial and forelimb movements that can be used to assess sensorimotor function in rodents.^16^, ^17^ On the second day following TBI surgery, the mice were placed in a new home cage containing five pieces of paper towel (50 × 70 mm, Weida) at 20:00. After 12 h, nest‐building ability was assessed using a scoring system ranging from 1 to 5, where 1 indicates intact and unmoved paper towels, 2 indicates scattered paper towels, 3 indicates lightly shredded paper towels gathered together, 4 indicates partially shredded paper towels without an obvious nest, and 5 indicates mostly shredded paper towels forming a nest around the mouse.

### 2.11 Elevated plus maze test

The apparatus for the EPMT height of 40 cm consisted of a central platform (5 cm × 5 cm), two open arms without walls (30 cm long and 5 cm wide), and two closed arms enclosed by 15‐cm‐high walls. All arms were extended from the central platform. The mice were placed at the central platform facing one of the closed arms and allowed to explore the maze individually for 5 min, while their behavior was recorded by a camera. The total time spent in the open arms and the number of entries into the open arms were calculated. The maze was cleaned with 75% ethanol between each trial.

### 2.12 Histology

The anesthetized mice were transcardially perfused with PBS. Brains were collected and immersed in 4% paraformaldehyde for more than 24 h. After 48 h of 30% sucrose dehydration, the brain was cut into 20‐μm‐thick slices by a cryotome (Leica).

For immunofluorescence histochemistry, brain sections were incubated with 0.5% Triton X‐100 for 30 min, blocked with 3% bovine serum albumin in PBS for 1 h, and then incubated overnight at 4°C with primary antibodies against BACE1 (1:200), NeuN (1:200), PSEN2 (1:200), and GABAbR1 (1:200). After three washes with PBS, the brain sections were incubated with 488 and 594 fluorescent secondary antibodies (1:400) for 1 h at room temperature. Subsequently, the sections were stained with DAPI for 10 min, washed three times with PBS, and mounted on slides.

For thioflavin T staining, according to a previous study,^18^ thioflavin T was prepared by dissolving 12.5 mg of dry powder in 100 mL 50% ethanol in ddH_2_O. Sections were then incubated in the thioflavin. For immunohistochemistry, brain sections were incubated with 0.5% Triton X‐100 and 1% H_2_O_2_ for 30 min to quench endogenous peroxidase activity. Subsequently, brain slices were blocked with 3% bovine serum albumin in PBS for 1 h. The sections were then incubated overnight at 4°C with primary antibodies against Aβ (Abcam, ab201060). Following three washes with PBS, the brain sections were incubated with a goat anti‐rabbit IgG (H + L) secondary antibody (1:200) for 1 h at room temperature. The staining was visualized using DAB (3,3′‐diaminobenzidine) for 3 min. Afterward, the sections underwent three washes with PBS. Following this, a stepwise dehydration process using ascending concentrations of alcohol and a clearance using xylene were performed. Finally, the sections were mounted on slides for further analysis.

For Nissl staining, brain sections were immersed in a staining solution containing cresyl violet (Beyotime, C0117, China) for 3 min. Following staining, the sections were thoroughly rinsed with water and subjected to a stepwise dehydration process using increasing concentrations of alcohol. Subsequently, the sections were cleared using xylene and mounted onto slides.

Images were taken by a fluorescence microscope (Mshot).

### 2.13 Electroencephalogram electrode implantation

Mice were implanted with electrodes for electroencephalogram (EEG) recording after TBI surgery. The exposed skull surface was dried with ethanol and attached to the base of a headmount (8201, Pinnacle) using cyanoacrylate. The headmount was positioned with its front edge 3 mm anterior of bregma, and four tapping screw holes were made using a 23‐gauge needle that was slowly rotated into the skull. For the anterior holes, 0.10 “screws (8209, Pinnacle) were used, while 0.12” screws (8212, Pinnacle) were used for the posterior holes. The screws were partially advanced using a screwdriver, and a small amount of silver epoxy (8226, Pinnacle) was applied to the outermost corner of each screw. All screws were then tightened down, and dental acrylic was applied.

### 2.14 EEG recording and analysis

The EEG recording system (8200‐K1, Pinnacle) was connected to the mouse headmounts on the second day after surgery, and mice were placed in a recording cage. EEG recording was conducted at 8 a.m. and continued for 24 h, while the mice were freely moving. The light phase is 8 am to 8 pm, and the dark phase is 8 pm to 8 am. The power spectral density of EEG data was performed using EEGLAB^19^ within MATLAB (Mathworks). A background noise filter with high‐frequency set at 0.45 Hz was used. The power spectral density for each frequency bin was presented from 0.5 to 60 Hz: (1) delta: 0.5–4 Hz, (2) theta: 4–8 Hz, (3) alpha: 8–12 Hz, (4) beta: 12–30 Hz, and (5) gamma: 30–60 Hz. The events and duration of seizures were quantified by SIRENIA SEIZURE PRO (Pinnacle) with the following criteria: at least twice the amplitude of the baseline EEG signal and two abnormal events separated by more than 0.5 s or counted as one.

### 2.15 Aβ clearance

To assess the clearance of Aβ,^20^ human Aβ42 was administered into the prefrontal cortex at coordinates 2 mm posterior, 1 mm lateral, and 1 mm deep (relative to bregma) on Day 3 after TBI surgery. The mice were anesthetized using pentobarbital sodium (50 mg/kg, intraperitoneal injection) and secured in a stereotactic instrument. Human Aβ42 (ChinaPeptides Co., Ltd), 1 mg dissolved in 1 mL of DMSO, was diluted to a concentration of 0.0451 mg/L using a 0.9% saline solution. A volume of 0.5 μL of the Aβ42 solution was injected into the left prefrontal cortex using a micropump at a rate of 0.1 μL/min. After the injection, the syringe was left in place for 10 min to allow optimal diffusion of Aβ42 into the brain parenchyma. One hour following the injection, the mice were euthanized by neck cutting, and brain tissue was collected. The levels of human Aβ42 in the mouse brain homogenates were subsequently measured using enzyme‐linked immunosorbent assay (ELISA) (E‐EL‐H0543c, Elabscience).

### 2.16 ELISA

ELISA was utilized for the quantitative determination of human Aβ42 in mouse brain homogenates and cell culture supernatant (Invitrogen, KMB3441). All reagents and samples were equilibrated to room temperature for 30 min. Subsequently, 100 μL of each standard solution or sample was added to the wells. The plate was then covered and incubated at 37°C for 1.5 h. After removing the cover, the solution was discarded, and 100 μL of the biotin‐detection antibody work solution was added to each well. The plate was covered again and incubated at 37°C for 1 h. Following this, the solution was discarded, and the wells were thoroughly washed three times with 350 μL of wash buffer. Next, 100 μL of the SABC (Streptavidin–Biotin–ABC) working solution was added to each well, and the plate was covered and incubated at 37°C for 30 min. After discarding the solution, the wells underwent five washes with wash buffer. Then, 90 μL of the TMB (3,3′,5,5′‐Tetramethylbenzidine) substrate was added to each well, and the plate was covered and incubated at 37°C in the dark for 20 min. Finally, 50 μL of the stop solution was added to each well, and the results were read at 450 nm within 20 min.

### 2.17 Quantitative real‐time PCR assays

RNA was isolated from HT22 cells and brains using Trizol reagent (R401, Vazyme). The isolated RNA was quantified using an Ultraspec 1000 system (Amersham Pharmacia Biotech). cDNA was then synthesized by using a First Strand cDNA Synthesis Kit (R211, Vazyme). qRT‐PCR was performed using a Quantstudio system (Thermo Fisher Scientific) with SYBR qPCR Master Mix (Q711, Vazyme) and specific primers. The primers utilized in this study: mouse ADAM10, F: 5′‐GGGAAGAAATGCAAGCTGAA‐3′, R: 5′‐CTGTACAGCAGGGTCCTTGAC‐3′; mouse BACE1, F: 5′‐TTCATCAACGGCTCCAACT‐3′, R: 5′‐CTCCAGGGAGTCGTCAGG‐3′; mouse PSEN2, F: 5′‐ACACAGAGCAGAGCCAAATCAAAGG‐3′, R: 5′‐CAGGGGAATGGTGAAGGAGAGGTAG‐3′; mouse GAPDH, F: 5′‐TCACCAGGGCTGCCATTTGC‐3′, R: 5′‐GACTCCACGACATACTCAGC‐3′. The mRNA levels were normalized to the standard housekeeping gene (GAPDH). The relative mRNA expression level of ADAM10, BACE1, and PSEN2 was calculated using the 2_T_^‐△△C^ method.

### 2.18 Western blot

Cells were harvested at 24 h after injury and lysed using 100 μL RIPA buffer (G2002‐100, Servicebio).

Mice were perfused with PBS 3 days after TBI, and half of the brain tissue containing the damaged area was quickly separated on ice. Brain tissues were homogenized in RIPA lysis buffer (10 μL/mg). The RIPA buffer contains an EDTA‐free protease inhibitor cocktail (G2006, Servicebio) and 1 mM PMSF (G2008, Servicebio). The lysates were clarified by centrifugation at 12 000 × *g* for 15 min at 4°C. The protein contents in the supernatant were measured using a bicinchoninic acid assay kit (G2026‐200T, Servicebio). The protein samples were fractionated by 8%–12% sodium dodecyl sulfate polyacrylamide gel and electrotransferred onto PVDF membranes (Merck Millipore). The membranes were blocked with 5% skimmed milk at room temperature for 1 h and then incubated overnight at 4°C with specific primary antibodies against β‐actin (1:2000), ADMA10 (1:1000), BACE1 (1:1000), PSEN2 (1:1000), PLCB3 (1:1000), p‐PLCB3 (1:1000), PLCG1 (1:1000), p‐PLCG1 (1:1000), NR1 (1:1000), NR2B (1:1000), GRIA1(1:1000), GRIA2 (1:1000), and GABAbR1 (1:1000). After three washes with PBS, the membranes were incubated with horseradish peroxidase‐conjugated secondary antibodies (1:5000) for 1 h at room temperature. After three washes with PBS, protein expression was detected using electrochemiluminescence and an imaging system (Tanon 5200), and quantified by ImageJ.

### 2.19 Statistical analysis

The statistical analyses were carried out using GraphPad Prism 8. All data are expressed as the mean ± SEM and tested with normal (Gaussian) distribution. Kruskal–Wallis test was utilized for between‐group comparisons due to the non‐normal distribution of data in ELISA and EEG analyses. For the remaining data, one‐way analysis of variance was employed for between‐group comparisons after passing the normality test, followed by Tukey's test. *p* < 0.05 was considered statistically significant.

## 3 RESULTS

### 3.1 ExD17 inhibits APP amyloidogenic cleavage and abnormal neural activity in a HT22 cell injury model

**FIGURE 2:**
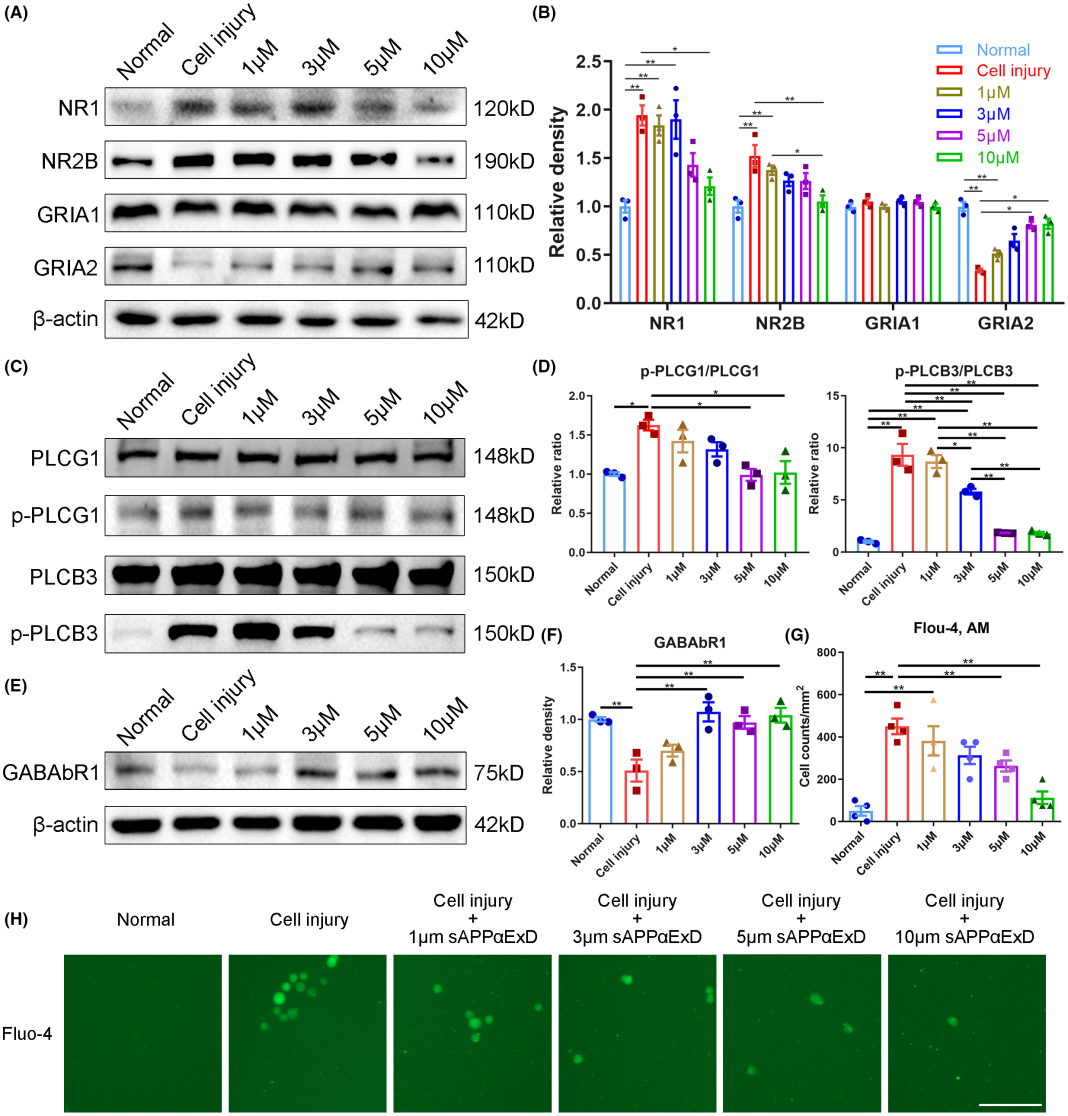
ExD17 inhibits abnormal neural activity in the model of HT22 cell injury. (A–F) Western blots and statistical analysis of GABAbR1, NR1, NR2B, GRIA1, GRIA2, p‐PLCG1, PLCG1, p‐PLCB3, and PLCB3 were performed after cell injury and treatment with various concentrations of ExD17. The ratios of p‐PLCG1/PLCG1 and p‐PLCB3/PLCB3 were analyzed in (D). *N* = 3 wells per group. (G, H) Representative images of Fluo‐4‐stained intracellular calcium‐positive cells and statistical analysis were performed. *N* = 4 wells per group. Scale bar = 100 μm. Values are presented as means ± SEM, *p < 0.05, **p < 0.01.

We first investigated the impact of ExD17 on a HT22 cell injury model. After inducing moderate stretch, the cell viability decreased by 50% 24 h postinjury. However, when ExD17 was administered immediately after stretching, the cell viability increased in a concentration‐dependent manner. At 1, 3, 5, and 10 μM, the corresponding cell viability rates were 55%, 62.5%, 73.7%, and 92.2%, respectively, 24 h after injury (Figure 1B). Following stretch injury, the transcription and translation of β‐ and γ‐secretase (BACE1 and PSEN2) were activated, while there was no change in α‐secretase (ADAM10). ExD17 treatment at varying concentrations induced a shift in the changes in β‐ and γ‐secretase (Figure 1D–F). The increase in Aβ42 level in the supernatant following cell injury was in line with the Western blot and PCR findings. In contrast, ExD17 treatment led to a significant reduction in Aβ42 level (Figure 1C). As ExD17 modulates synaptic transmission by binding to the sushi domain of GABAbR1 and inhibits neural activity, we examined the effect of ExD17 on the expression of GABAbR1 and synaptic function in the cell injury model. Our findings showed that cell injury caused a considerable decrease in GABAbR1 expression, which was restored to normal levels by treatment with ExD17 at concentrations of 3, 5, or 10 μM (Figure 2E,F). Following cell injury, GRIA2, the postsynaptic AMPA receptor, showed a significant reduction in its level, which was restored upon treatment with ExD17 at concentrations of 5 or 10 μM for 24 h. Conversely, the level of GRIA1 remained unchanged. Additionally, NMDA receptor subunits NR1 and NR2B increased after injury but returned to normal levels following treatment with 10 μM ExD17 (Figure 2A,B).

These results suggested that the increase in neural activity could be attributed to the reduction of GABAb receptors and the elevation of NMDA receptors. To further test this hypothesis, we conducted the calcium imaging experiment using Fluo‐4 AM dye to quantify intracellular Ca^2+^ levels. The number of Fluo‐4‐positive cells increased after cell injury, but the count reduced significantly in a concentration‐dependent manner when cells were treated with 3, 5, or 10 μM ExD17 (Figure 2G,H). We also measured the levels of intracellular Ca^2+^ using the activated forms of PLCG1 and PLCB3. The ratios of p‐PLCG1/PLCG1 and p‐PLCB3/PLCB3 were increased after cell injury, indicating elevated intracellular Ca^2+^ levels. However, treatment with ExD17 reduced these ratios in a concentration‐dependent manner (Figure 2C,D).

### 3.2 The topical application of ExD17 effectively inhibits APP amyloidogenic cleavage, but it does not provide protection against cognitive impairment in mice with TBI

**FIGURE 3:**
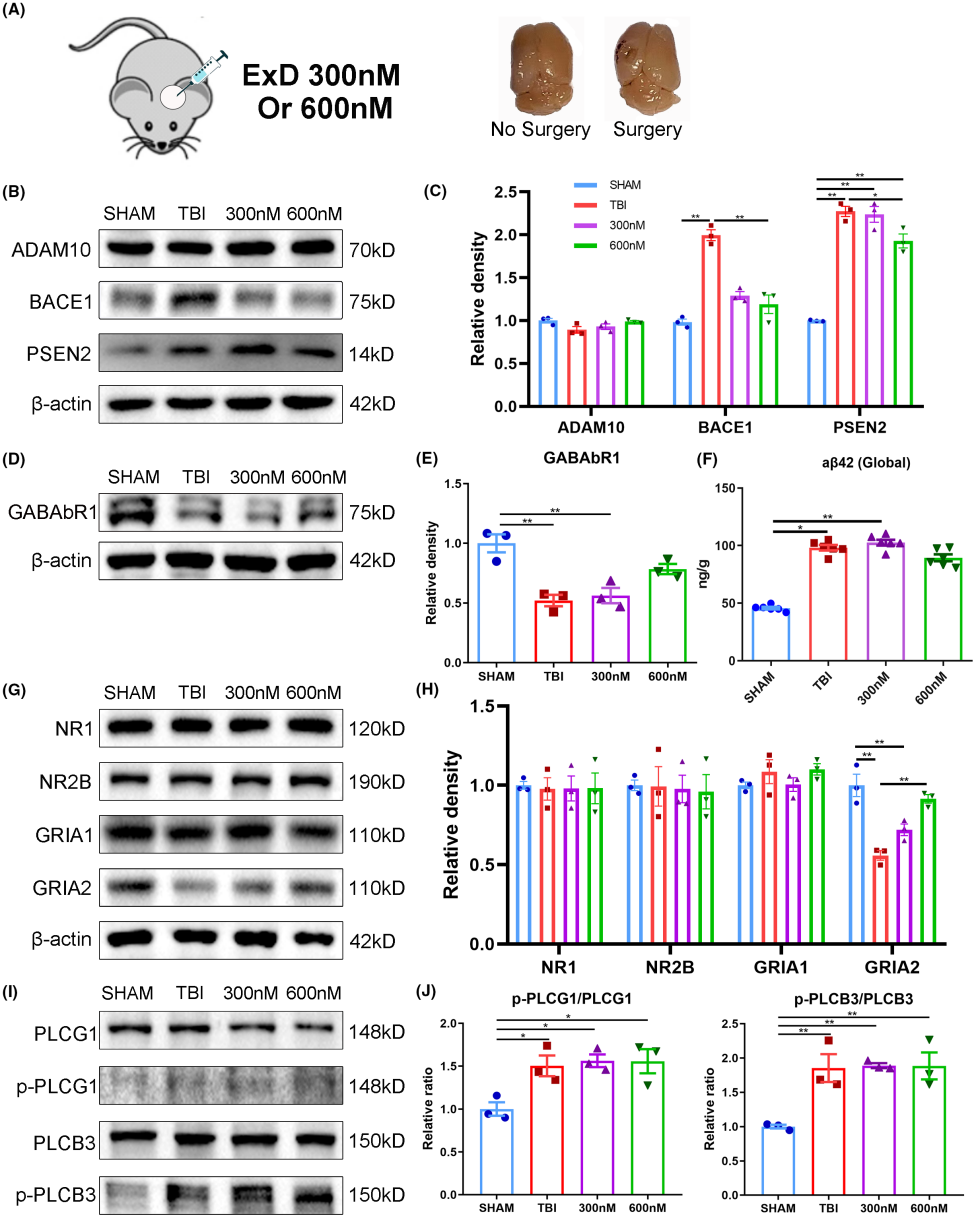
Topical administration of ExD17 inhibits APP amyloidogenic cleavage. (A) Schematic of the topical administration of ExD17 and the comparison of brains after surgery. (B–E) Western blots and statistical analysis of ADAM10, BACE1, PSEN2, and GABAbR1 in the sham, TBI, and TBI topically treated with 300 or 600 nM ExD17 groups. *N* = 3 per group. (F) Levels of Aβ42 in the cortex of sham, TBI, and TBI topically treated with 300 or 600 nM ExD17, were analyzed using ELISA on Day 3 after TBI surgery. *N* = 3 per group. G–J. Western blots and statistical analysis of NR1, NR2B, GRIA1, GRIA2, p‐PLCG1, PLCG1, p‐PLCB3, and PLCB3 in the sham, TBI, and TBI topically treated with 300 or 600 nM ExD17 groups. In Panel (J), the ratios of p‐PLCG1/PLCG1 and p‐PLCB3/PLCB3 are analyzed. *N* = 3 per group. Values are presented as means ± SEM, *p < 0.05, **p < 0.01.

**FIGURE 4:**
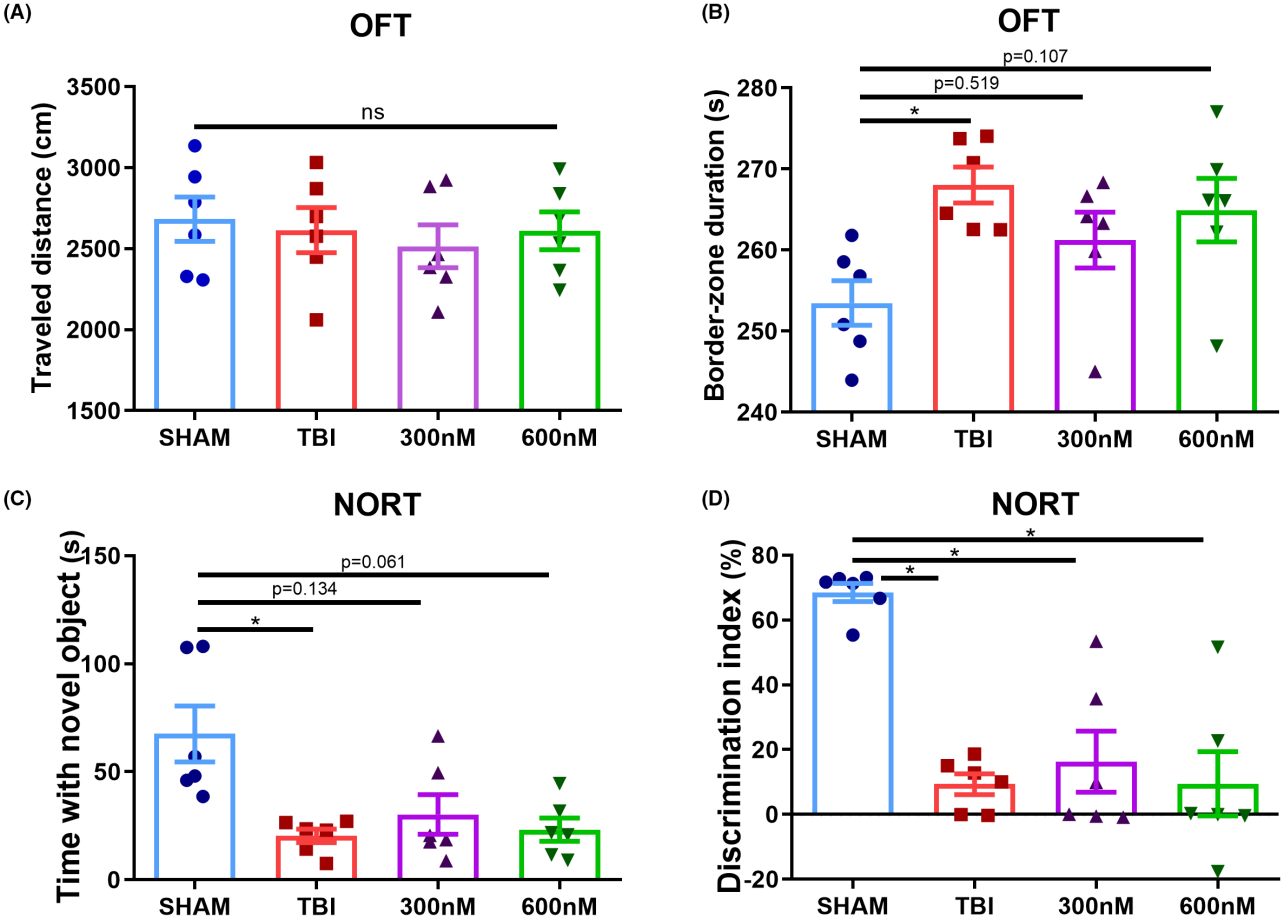
Topical administration of ExD17 cannot improve behavioral outcomes. (A, B) The total distance traveled and the duration of border zones in the OFT were measured on Day 3 after surgery. *N* = 6 per group. (C, D) The time spent exploring the novel object and the discrimination index of the novel object recognition test were measured on Day 3 after surgery. *N* = 6 per group. Values are presented as means ± SEM, *p < 0.05.

As observed the protective effects of ExD17 on injured cells in vitro, we sought to investigate its potential effects in vivo. After TBI surgery, we administered ExD17 topically on the cortical surface at a concentration of 300 or 600 nM and collected the brains 3 days postsurgery (Figure 3A). Consistent with the results of in vitro experiments, we observed an increase in APP amyloidogenic cleavage after TBI surgery, as demonstrated by the significant upregulation of BACE1 and PSEN2 expression. However, the administration of ExD17 resulted in a return to normal levels. The expression of ADAM10 was unaffected across all groups, indicating that ExD17 selectively inhibits the amyloidogenic pathway without affecting the nonamyloidogenic pathway (Figure 3B,C). In line with the findings on secretases, Aβ42 production elevated following TBI but was not normalized by ExD17 administration (Figure 3F). Moreover, the expression of GABAbR1 reduced after TBI and ExD17 treatment (Figure 3D,E). The expression of GRIA2 decreased following TBI, but could be increased with treatment at a concentration of 600 nM (Figure 3G,H). Furthermore, the ratios of p‐PLCG1/PLCG1 and p‐PLCB3/PLCB3 increased after TBI but did not differ after ExD17 treatment (Figure 3I,J). The results indicated that administering ExD17 topically after TBI with a single dose was sufficient to inhibit Aβ processing. However, it was not able to effectively suppress neuronal hyperactivity during the 3‐day period, as fewer changes were observed in the synaptic proteins. Subsequently, the behaviors of the mice were then tested by OFT and NORT 3 days after surgery. The OFT results indicated a higher number of peripheral zones than the animals in the sham group, indicating that anxiety was induced after TBI. Nevertheless, the administration of ExD17 did not eliminate anxiety in TBI mice (Figure 4A,B). TBI mice spent less time with the novel object and had a lower preference index compared to sham animals, and cognitive dysfunction was not rescued by the administration of ExD17 (Figure 4C,D).

### 3.3 Repeated ExD17 administration suppresses Aβ processing in TBI mice

**FIGURE 5:**
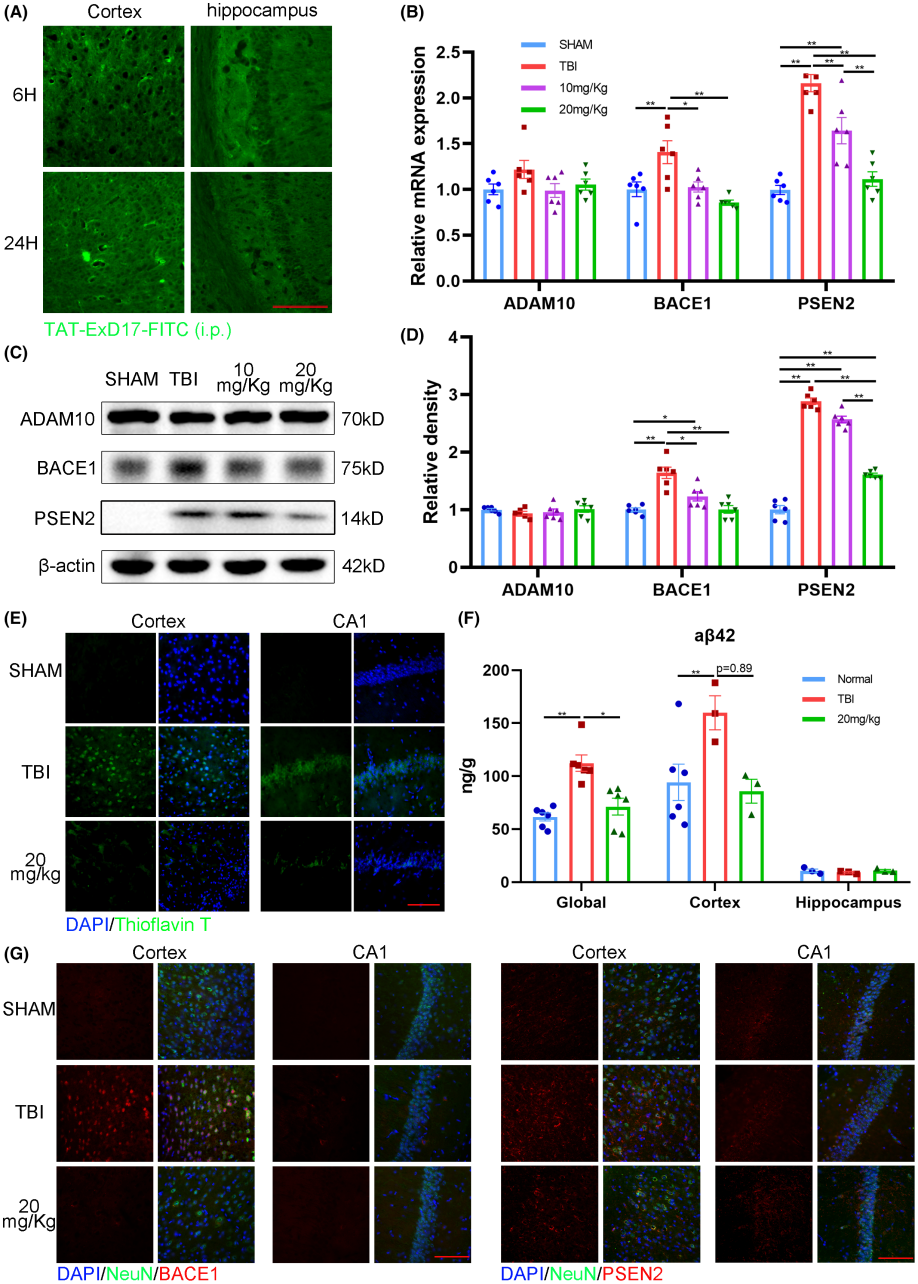
Repeated ExD17 administration suppresses Aβ processing in TBI mice. (A) TAT‐ExD17‐FITC (20 mg/kg) was injected i.p. into wild‐type mice. The fluorescent signal of FITC in the brains was detected 6 or 24 h after injection. Scale bar = 10 μm. (B) RT‐PCR was used to determine the mRNA expression levels of ADAM10, BACE1, and PSEN2 3 days after surgery and ExD17 treatment. *N* = 6 per group. (C, D) Western blots and statistical analysis of ADAM10, BACE1, and PSEN2 3 days after surgery and ExD17 treatment. *N* = 6 per group. (E) Representative images of thioflavin T staining in the cortex and CA1 of hippocampus. Scale bar = 10 μm. (F) ELISA was used to analyze the levels of Aβ42 in the whole brain, cortex, and hippocampus among the groups. *N* = 3 per group. (G) Representative images of the expressions of BACE1 and PSEN2 in the cortex and CA1. Scale bar = 10 μm. Values are presented as means ± SEM, *p < 0.05, **p < 0.01.

A reasonable assumption was that a single dose of ExD17 would not provide sustained protection. To address this, we developed a TAT‐ExD17 peptide (Figure 1A), which could potentially cross the blood–brain barrier (BBB) via the TAT motif following intraperitoneal (i.p.) injection. To assess the effectiveness of peptide crossing of the BBB, we i.p. injected TAT‐ExD17‐FITC (20 mg/kg) into wild‐type mice. After 6 and 24 h, robust FITC signals were observed in the cortex and hippocampus, indicating that the peptides effectively crossed the BBB (Figure 5A).

**FIGURE 6:**
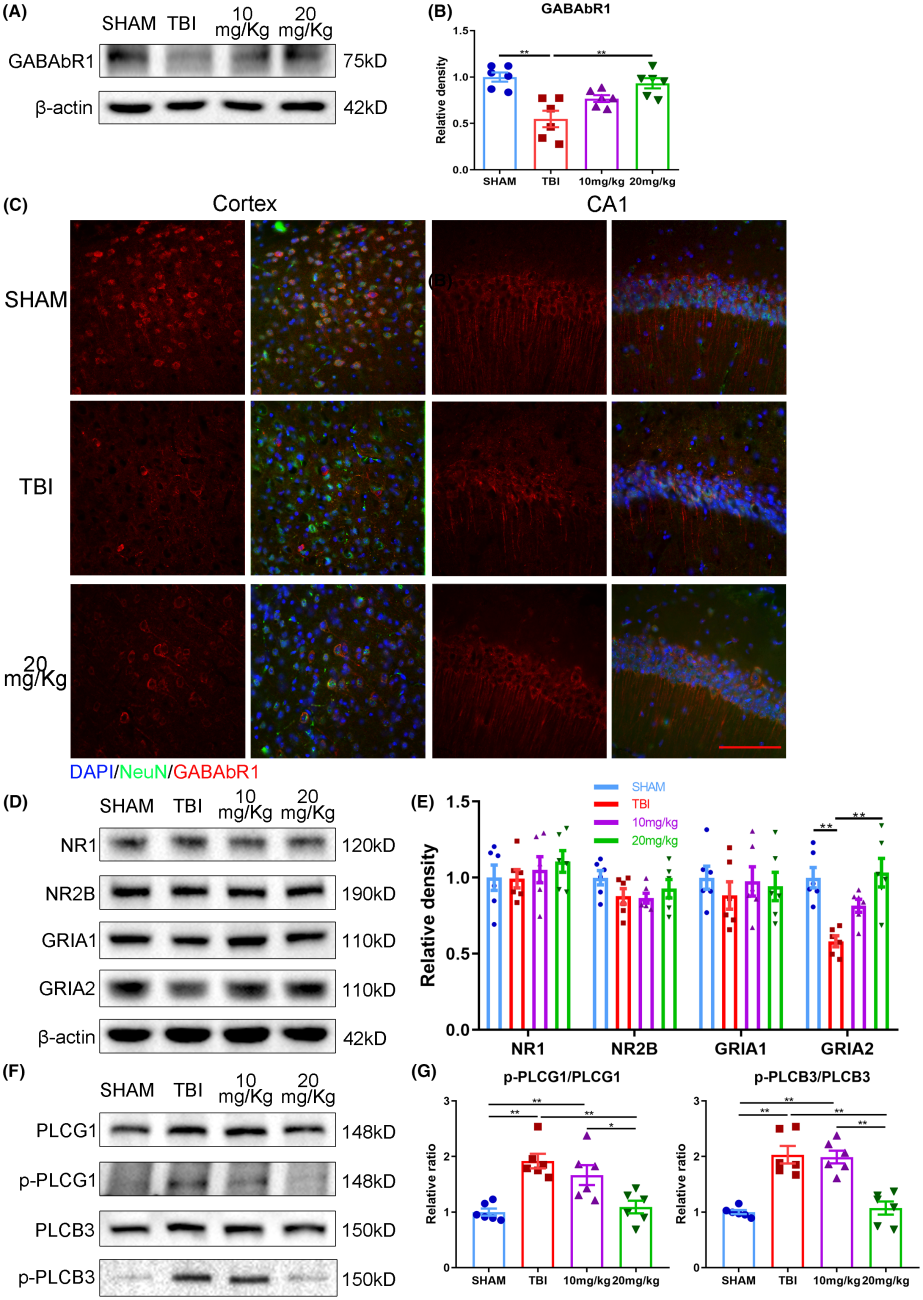
Repeated ExD17 administration inhibits abnormal neuronal activity in TBI mice. (A, B) Western blots and statistical analysis of GABAbR1 3 days after surgery and ExD17 treatment. *N* = 6 per group. (C) Representative images of GABAbR1 expression in the cortex and CA1 3 days after surgery and ExD17 treatment. Scale bar = 10 μm. (D–G) Western blots and statistical analysis of NR1, NR2B, GRIA1, GRIA2, p‐PLCG1, PLCG1, p‐PLCB3, and PLCB3 in the sham, TBI, and TBI treated with 10 or 20 mg/kg ExD17 groups. In Panel (G), the ratios of p‐PLCG1/PLCG1 and p‐PLCB3/PLCB3 are analyzed. *N* = 6 per group. Values are presented as means ± SEM, *p < 0.05, **p < 0.01.

**FIGURE 7:**
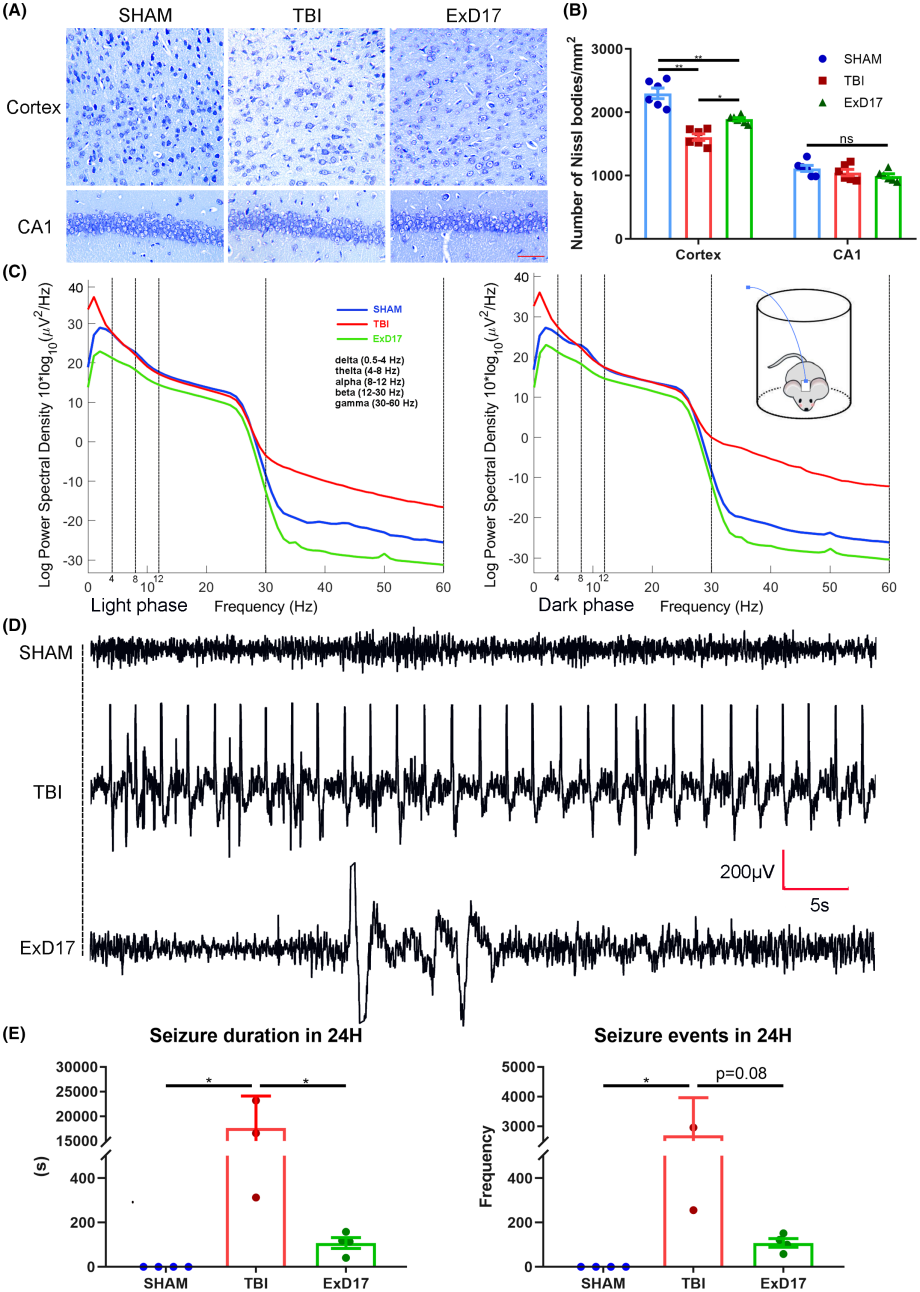
Repeated ExD17 administration inhibits abnormal neuronal activity in TBI mice. A. Representative images of Nissl staining in the cortex and CA1 3 days after surgery and ExD17 treatment. Scale bar = 50 μm. B. Statistical analysis of neuronal survival in the cortex and CA1 in the three groups. N = 6 per group. C. EEG spectral power plots in the light and dark phases in the sham, TBI, and TBI treated with ExD17 groups. D. Representative EEG traces from the three groups. E. Seizure duration and total seizure events in the sham, TBI, and TBI treated with ExD17 groups. N = 4 per group. Due to the truncation of the y‐axis, some individual data for the TBI group were not displayed. Values are presented as means ± SEM, *p < 0.05, **p < 0.01.

The effects of ExD17 were evaluated by administering TAT‐ExD17 (10 or 20 mg/kg) for 3 days via i.p. injection after TBI surgery. Similar to the findings with topical ExD17 administration, TBI resulted in a decrease in the expression of GABAbR1, which was restored to normal levels with the administration of 20 mg/kg TAT‐ExD17 (Figure 6A,B). Immunofluorescence histochemistry results showed that GABAbR1 was upregulated throughout the brain, including the cortex and hippocampus (Figure 6C). Furthermore, the administration of 20 mg/kg TAT‐ExD17 resulted in a decrease in the expression level of BACE1 and PSEN2 compared to the TBI group, indicating inhibition of Aβ processing. However, the expression of ADAM10 was not affected by ExD17 administration (Figure 5B–D). The changes in BACE1 and PSEN2 were also confirmed by immunofluorescence histochemistry (Figure 5G). Consequently, TBI mice exhibited a significant increase in cortical Aβ42 levels, which returned to normal after the administration of 20 mg/kg TAT‐ExD (Figure 5F). Amyloid plaques, which were deposited in the cortex and hippocampus of TBI mice, were reduced after 20 mg/kg TAT‐ExD administration, as confirmed by thioflavin T staining (Figure 5E and Figure S1A). To further verify the reduction of Aβ after the TAT‐ExD treatment, attributed to the enhanced ability of Aβ clearance, we administered human Aβ42 into the brain and conducted an analysis of residual substances using ELISA after a 30‐min circulation period.^20^ The findings indicated comparable levels of residual substances in the brain across all groups, suggesting a similar rate of Aβ clearance among the groups (Figure S1B). Furthermore, the loss of neurons in the TBI group was not detected in the 20 mg/kg ExD17 group (Figure 7A,B).

### 3.4 Repeated ExD17 administration inhibits abnormal neuronal activity in TBI mice

ExD17 interacts with GABAbR1, thereby modulating presynaptic vesicle release and leading to the inactivation of GABAergic interneurons.^10^ Given this mechanism, it is reasonable to assume that ExD17 may improve TBI outcomes by inhibiting neuronal activity. We assessed the expression of synaptic proteins, which revealed a decrease in GRIA2 levels in TBI mice. However, GRIA2 levels returned to normal after the administration of 20 mg/kg ExD17 (Figure 6D,E). Contrary to the findings from topical administration, the ratios of p‐PLCG1/PLCG1 and p‐PLCB3/PLCB3 increased after TBI but were reduced after administering 20 mg/kg ExD17 (Figure 6F,G). To further investigate the effect of ExD17 on neuronal activity, we implanted an EEG detector on the skull and recorded a 24‐h EEG on the third day after surgery. TBI mice had higher EEG power in delta and gamma frequencies than sham and ExD17 mice in both light and dark phases of freely moving mice. In contrast, TBI mice that received 20 mg/kg ExD17 exhibited significantly lower EEG power across all frequencies than sham and TBI mice (Figure 7C). The number of seizure events in the TBI group was higher and longer than in the sham group. In comparison, the number of seizure events in mice treated with 20 mg/kg ExD17 was significantly reduced, and the duration of seizures was significantly shortened (Figure 7D–F).

### 3.5 Repeated administration of ExD17 improves cognitive outcomes in TBI mice

**FIGURE 8:**
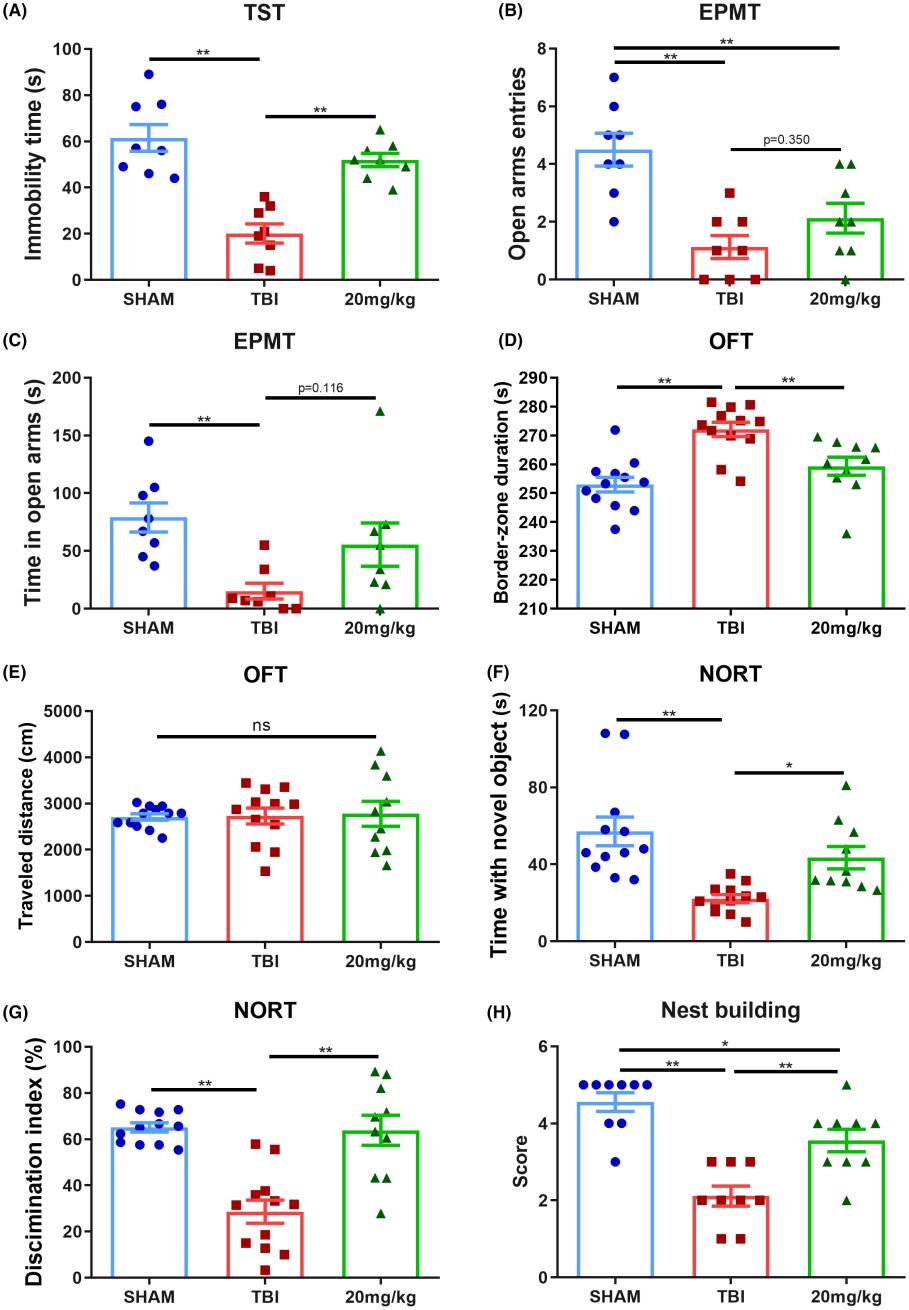
Repeated administration of ExD17 improves the behavioral outcomes of TBI mice. (A) The immobility time in the TST was measured in all groups. *N* = 8 per group. (B, C) The duration and number of entries into the open arms in the EPMT were measured. *N* = 8 per group. (D and E) Total distance traveled and the duration of border zones in the open field were measured 3 days after surgery. N (sham) = 12, N (TBI + vehicle) = 12, N (TBI + ExD17) = 10. (F, G) Time spent exploring the novel object and the discrimination index of the NORT were measured 3 days after surgery. N (sham) = 12, N (TBI + vehicle) = 12, N (TBI + ExD17) = 10. (H) Nest‐building activity was scored based on the complexity of the construction: 1 = paper towels are intact and have not moved, 2 = paper towels scattered throughout the cage, 3 = paper towers little shredded and gathered, 4 = paper towels partially shredded but no obvious nest, 5 = paper towels mostly shredded into pieces and surround the mouse. *N* = 9 per group. Values are presented as means ± SEM, *p < 0.05, **p < 0.01.

These data suggested that the administration of 20 mg/kg ExD17 had a remarkable effect in preventing amyloidogenic cleavage and suppressing abnormal neural activities. We proceeded to evaluate the effect of 20 mg/kg ExD17 on animal behaviors. TBI mice showed a higher desperation, as evidenced by longer immobility periods in the TST (Figure 8A), reduced entries and shorter duration in the open arms of the EPMT (Figure 8B,C), and increased border zone crossings in the OFT (Figure 8D,E) compared to the sham group. The administration of ExD17 alleviated the despair‐like behavior.

The administration of ExD17 resulted in an extension of time spent on the novel object in the NORT, which was shortened in TBI mice. The discrimination of novel objects was impaired in TBI mice, as evidenced by a decreased preference index, which was similar between the sham and ExD17 groups, indicating that ExD17 administration prevented TBI‐induced cognitive dysfunction (Figure 8F,H). In addition, the nesting abilities were poor in TBI mice but were restored by ExD17 administration (Figure 8G).

## 4 DISCUSSION

Our study demonstrates that ExD17, the extension domain of sAPPa, improves the outcomes of TBI by inhibiting the amyloidogenic cleavage pathway of APP and suppressing neural activities in a cell injury model and a TBI mouse model. Previous studies have shown that sAPPα can exert neurotrophic and neuroprotective functions by inhibiting APP amyloidogenic cleavage. sAPPα binds to the allosteric site of BACE1, attenuating Aβ pathology,^21^, ^22^ and can also transport Aβ across the BBB via endothelial LRP1 to remove Aβ from the brain to the periphery.^23^ In rats, the administration of the heparin binding site of sAPPα (residues 96–110) has been shown to reduce hippocampal neuronal death against TBI.^24^, ^25^ Our findings reveal that ExD17, which is the GABAb binding domain of sAPPα (204–220), effectively suppressed the expression of β‐secretase and γ‐secretase (PSEN2) and reduced the production of Aβ. However, ExD17 did not have a significant effect on the expression of α‐secretase, indicating that its mechanism of action involves the inhibition of APP amyloidogenic processing.

GABAergic interneurons are lost after TBI, leading to a reduction of synaptic inhibition and an increase in hippocampal excitability.^26^ The GABAb receptors are G‐protein‐coupled receptors present on both presynaptic and postsynaptic membranes.^27^ These receptors produce multiple effects, such as reducing presynaptic neurotransmitter release and generating postsynaptic inhibitory K^+^ currents that hyperpolarize the membrane and inhibit neuronal activity. Rice's research indicated that ExD17 can bind to the sushi domain of GABAbR1a and suppress presynaptic transmission, thereby inhibiting neural activity.^10^ Therefore, targeting the GABAb receptor using ExD17 may provide a potential therapy for TBI by suppressing cerebral excitability.

Our findings demonstrate that ExD17 administration increases the expression of GABAb receptors and suppresses neural excitability in both TBI mouse model and cell injury model. The inhibition of neural activity caused by ExD17 may be due to the modulation of postsynaptic glutamate receptors and inhibition of intracellular Ca^2+^ concentrations. Specifically, stretch‐induced cell injury results in a decrease in GluR2 and an increase in NR1 and NR2A after 24 h. However, the administration of ExD17 reverses these changes. Level of GluR2 is significantly reduced after TBI but returned to normal level with ExD17 treatment. However, there are no significant differences in *N*‐methyl‐d‐aspartate receptors (NMDARs) in animal study, suggesting that the timing of the tests may have played a role in the differences between the cell and animal results. In the animal TBI model, NMDA receptors change within 24 h.^28^, ^29^ Reduced GluR2 is mediated by NMDAR and allows for increased intracellular Ca^2+^ flux.^30^ The administration of ExD17 relieves the chaos of glutamatergic proteins and inhibits neural excitability. This is also evidenced by fewer epilepsy events and delta and gamma power in the cortex after ExD17 administration.

In addition, we find that the phosphorylated Ca^2+^‐associated proteins PLCG and PLCB increase after TBI and cell injury but return to normal levels after the administration of 20 mg/kg ExD17. PLCG1 plays a crucial role in signal transduction by catalyzing the hydrolysis of phosphatidylinositol‐4,5‐biophosphate, leading to the formation of inositol‐1,4,5‐triophosphate (IP3) and 1,2‐diacylglycerol (DAG). IP3 and DAG subsequently activate intracellular Ca^2+^ and protein kinase C (PKC) signaling pathways.^31^ Additionally, PLCB3 is involved in the production of IP3 and the subsequent release of Ca^2+^ from intracellular stores.^32^ Our study reveals that after TBI, PLCG1 and PLCB3 are significantly activated, leading to increased intracellular Ca^2+^ flux, which is likely to contribute to neuronal death. The short‐term and limited protective effects of topical ExD17 administration on TBI prompt us to design the TAT‐ExD17 peptide, which can easily cross the BBB via i.p. injection and be repeatedly delivered. The administration of TAT‐ExD17 improves the prognosis of TBI and provides an effective treatment option. However, it should be noted that in these experiments, rather unphysiologically high concentrations of 300–600 nM ExD17 (topically) and 20 mg/kg (i.p.) were used, whereas the measured in vivo concentrations of sAPPα within the interstitial fluid are only approximately 1 nM.^33^ Furthermore, ExD17 demonstrates a decrease in the frequency of calcium transients, however, only at concentrations as high as 5 μM (Figure 2G,H). Furthermore, the investigation is necessary to explore the long‐term effects of ExD17 on cognitive function and epilepsy, as our experiments only encompassed a 3‐day treatment period following TBI.

In summary, our findings indicate that ExD17, the extension domain of APP, improves the outcomes of TBI and cell injury by inhibiting APP amyloidogenic cleavage and reducing abnormal neural activities.

## AUTHOR CONTRIBUTIONS

Xinghua Liu and Xiangjun Bai designed the experiments. Zhenxing Xie, Wei Su, Yanyun Lou, Tianyu Li, Xiyuan Zhou, and Yongsheng Zhang conducted the experiments and analyzed the data. Zhenxing Xie and Xinghua Liu wrote the manuscript. Xinghua Liu, Xiangjun Bai, and Zhanfei Li revised the manuscript. Xinghua Liu and Zhanfei Li supervised the project. All authors contributed to the article and approved the manuscript.

## CONFLICT OF INTEREST STATEMENT

The authors declare no conflicts of interest.

## Supporting information


Figure S1.

## Data Availability

The data used in this work are available from the corresponding author on reasonable request.
