# Supplementary material for: Extension domain of amyloid processor protein inhibits amyloidogenic cleavage and balances neural activity in a traumatic brain injury mouse model
Source: CNS Neurosci Ther. 2023 Aug 17;30(2):e14402. doi: 10.1111/cns.14402 (PMC10848085; doi:10.1111/cns.14402)
Supplement: Supplementary file 1 — Figure S1. [file CNS-30-e14402-s001.zip › FigureS1.pdf]

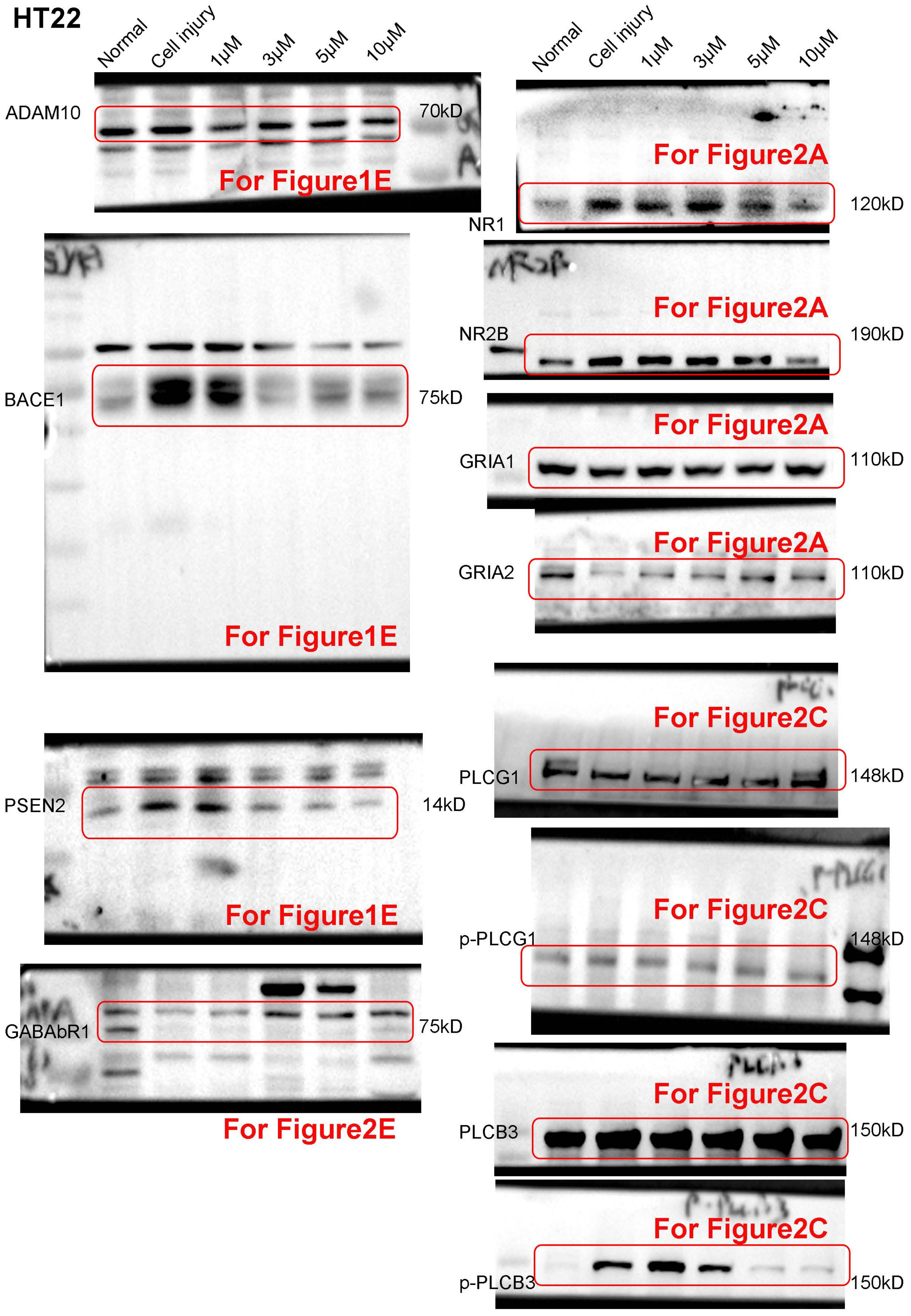
HT22
Normal
Cell injury
1μM
3μM
5μM
10μM
ADAM10
70kD
For Figure1E
BACE1
75kD
For Figure1E
PSEN2
14kD
For Figure1E
GABAbR1
75kD
For Figure2E
For Figure2A
NR1
120kD
For Figure2A
NR2B
190kD
For Figure2A
GRIA1
110kD
For Figure2A
GRIA2
110kD
For Figure2C
PLCG1
148kD
For Figure2C
p-PLCG1
148kD
For Figure2C
PLCB3
150kD
For Figure2C
p-PLCB3
150kD

## TBI local

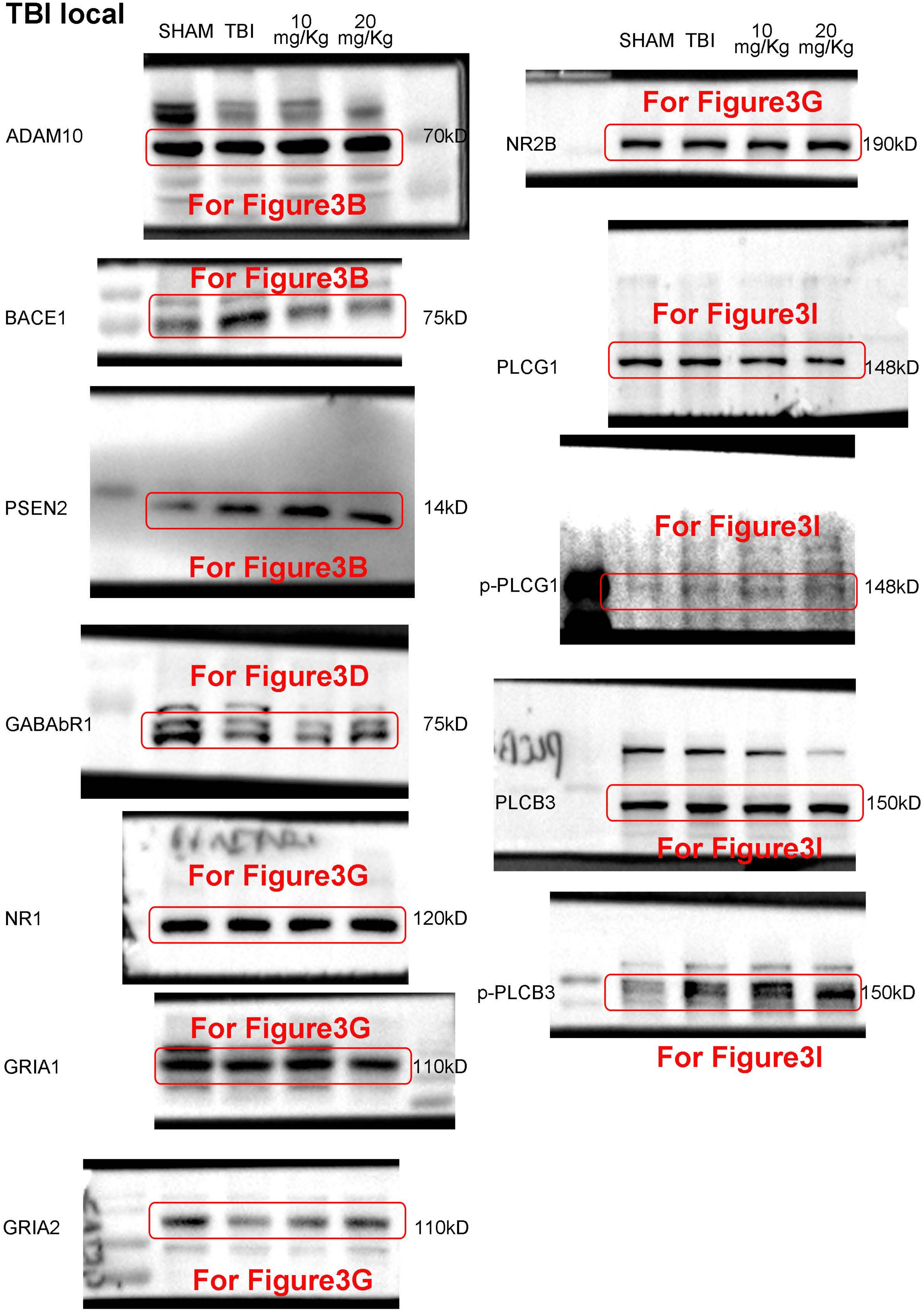
SHAM
TBI
10 mg/Kg
20 mg/Kg
ADAM10
70kD
For Figure3B
BACE1
75kD
For Figure3B
PSEN2
14kD
For Figure3B
GABAbR1
75kD
For Figure3D
NR1
120kD
For Figure3G
GRIA1
110kD
For Figure3G
GRIA2
110kD
For Figure3G
SHAM
TBI
10 mg/Kg
20 mg/Kg
NR2B
190kD
For Figure3G
PLCG1
148kD
For Figure3I
p-PLCG1
148kD
For Figure3I
PLCB3
150kD
For Figure3I
p-PLCB3
150kD
For Figure3I

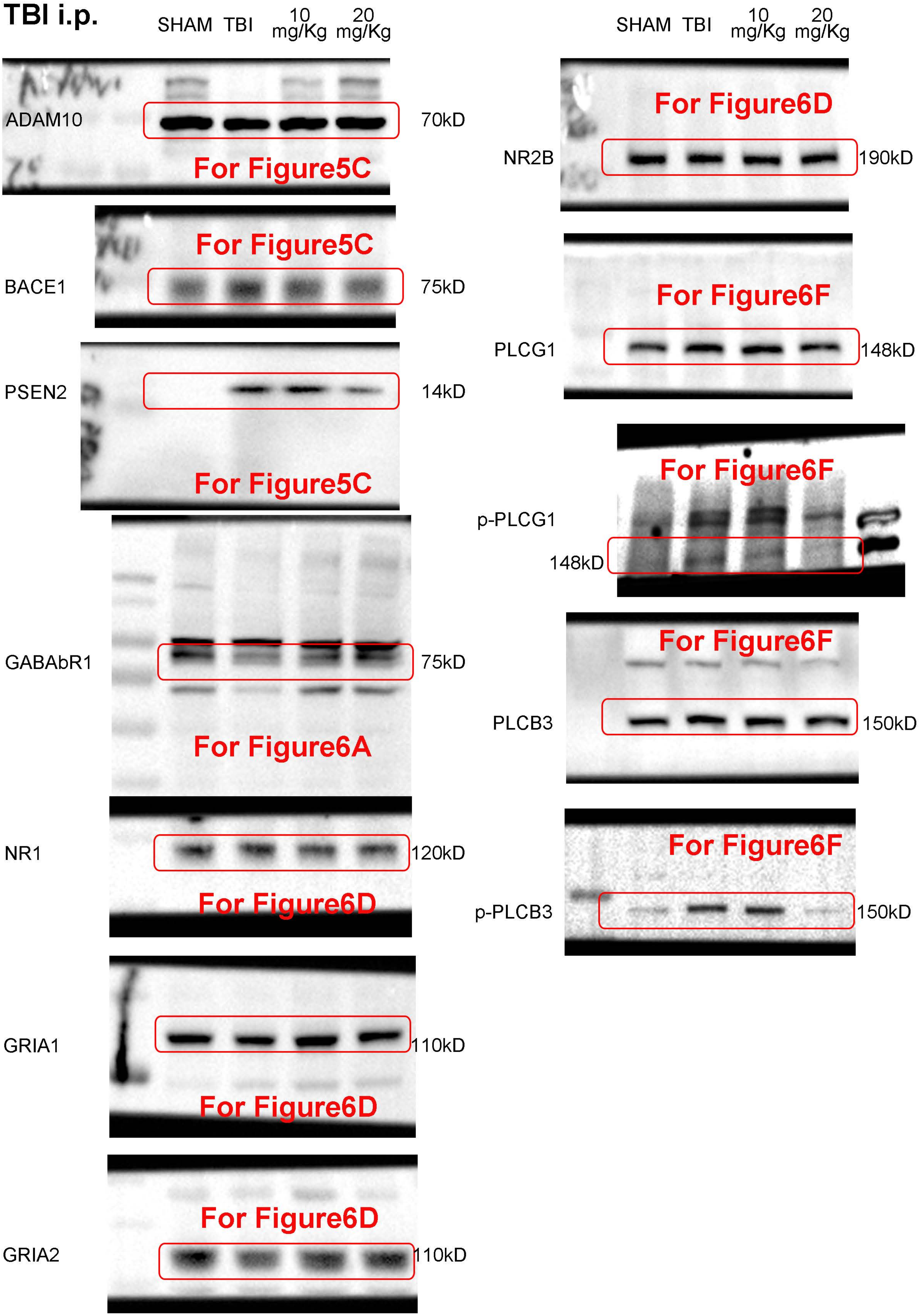
TBI i.p.
SHAM TBI 10 mg/Kg 20 mg/Kg
SHAM TBI 10 mg/Kg 20 mg/Kg
ADAM10
70kD
For Figure5C
BACE1
For Figure5C
75kD
PSEN2
14kD
For Figure5C
GABAbR1
75kD
For Figure6A
NR1
120kD
For Figure6D
GRIA1
110kD
For Figure6D
For Figure6D
GRIA2
110kD
For Figure6D
NR2B
190kD
For Figure6F
PLCG1
148kD
For Figure6F
p-PLCG1
148kD
For Figure6F
PLCB3
150kD
For Figure6F
p-PLCB3
150kD
