# Supplementary figures and images for: Extension domain of amyloid processor protein inhibits amyloidogenic cleavage and balances neural activity in a traumatic brain injury mouse model
Source: CNS Neurosci Ther. 2023 Aug 17;30(2):e14402. doi: 10.1111/cns.14402 (PMC10848085; doi:10.1111/cns.14402)

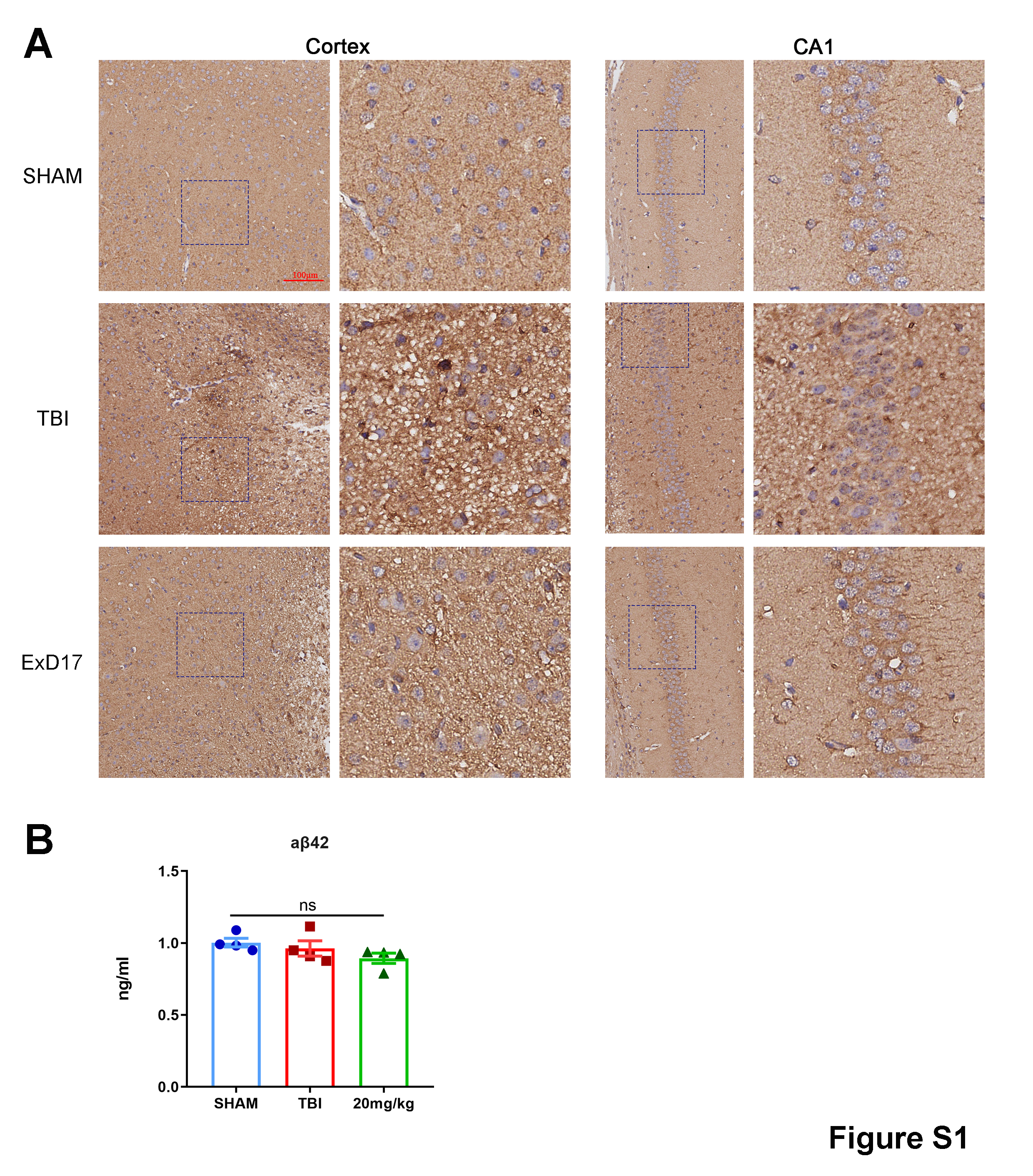

Supplement: Supplementary file 1 — Figure S1. [file CNS-30-e14402-s001.zip › Figure S1.tif]
